# Association between serum Klotho levels and the prevalence of diabetes among adults in the United States

**DOI:** 10.3389/fendo.2022.1005553

**Published:** 2022-11-09

**Authors:** Kai Wang, Yukang Mao, Miao Lu, Xianling Liu, Yan Sun, Zhongming Li, Yansong Li, Yinzhang Ding, Jing Zhang, Jian Hong, Di Xu

**Affiliations:** ^1^ Department of Geriatrics, The First Affiliated Hospital of Nanjing Medical University, Nanjing, Jiangsu, China; ^2^ Department of Cardiology, The First Affiliated Hospital of Nanjing Medical University, Nanjing, Jiangsu, China; ^3^ Department of Cardiology, The Affiliated Suzhou Hospital of Nanjing Medical University, Suzhou Municipal Hospital, Gusu School, Nanjing Medical University, Suzhou, Jiangsu, China

**Keywords:** Klotho, diabetes, adults, cross-sectional study, National Health and Nutrition Examination Survey (NHANES)

## Abstract

**Background:**

Diabetes is a critical contributor to the pathogenesis of cardiovascular diseases. Klotho is an anti−aging protein with cardiovascular-renal protective effects. However, the relationship between serum Klotho levels and diabetes remains poorly understood.

**Objectives:**

This study aimed to investigate the relationship between serum Klotho levels and diabetes in US adults.

**Methods:**

We analyzed the cross-sectional data obtained from 13751 subjects aged 40-79 years in the National Health and Nutrition Examination Survey (NHANES) (2007–2016). Serum Klotho concentration was measured using an enzyme-linked immunosorbent assay (ELISA) and categorized into four quartiles (Q1-Q4). Multivariate logistic regression and restricted cubic spline (RCS) regression were conducted to explore the association between serum Klotho levels and the prevalence of diabetes.

**Results:**

As compared with quartile 1, serum Klotho levels in quartiles 2-4 yielded odds ratios (OR) (95% CI) of diabetes of 0.96 (0.80–1.15), 0.98 (0.82–1.18), and 1.25 (1.04–1.50), respectively, after covariate adjustment (*P* for trend = 0.018). The results implied an increased risk of diabetes. The RCS plot showed a U-shaped relationship linking serum Klotho levels with diabetes (*P* for nonlinearity = 0.003).

**Conclusions:**

In summary, a nonlinear and positive association was found between serum Klotho levels and the prevalence of diabetes. Further study is needed to verify the causality of this association and elucidate the underlying mechanisms.

## Introduction

Diabetes has become a major health concern over the past few decades, placing a significant burden on healthcare systems over the world. It is also increasingly being recognized as an independent risk factor for various cardiovascular and renal disorders (1). Diabetes and its cardio-renal complications will likely be the leading causes of disability and mortality in the future (2). The incidence of diabetes in the elderly population worldwide is increasing dramatically, having reached 135.6 million in 2019 and being predicted to double by 2045, according to a recent International Diabetes Federation report (3). Therefore, a comprehensive understanding of the underlying mechanisms of diabetes is of utmost significance.

Klotho (generally referred to simply as α-Klotho) is encoded by the Klotho gene and was originally described to possess anti-aging properties. Klotho deficiency in mice resulted in a shortened lifespan of 8-9 weeks and induced a series of complications including premature aging, infertility, vascular calcification, arteriosclerosis, skin atrophy, osteoporosis and emphysema (4). In contrast, Klotho overexpression extended life expectancy and exerted therapeutic effects (5). Typically, Klotho content decreases during aging, and reduced Klotho expression has been consistently noted in various pathological conditions, including Alzheimer’s disease, chronic obstructive pulmonary disease (COPD), chronic renal disease (CKD), cardio-cerebrovascular disease and diabetes, which are all highly related to aging (6, 7). Numerous studies involving both genetic and experimentally-induced animal models of diabetes suggest the significant cardio-renal benefits of Klotho (8–11). Early vascular aging and endothelial dysfunction are hallmarks of cardio-renal disease in diabetes, and are highly related to upregulated renal expression and decreased serum and urine Klotho levels (12). Likewise, reduced serum Klotho levels were strongly linked to a faster rate of decline in estimated glomerular filtration rate (eGFR) in diabetic patients, implying that reduced Klotho levels may predict diabetes-associated renal impairment (13, 14). In a prospective study including 107 patients with diabetic nephropathy, low serum Klotho levels were linked to a higher risk of cardiac hypertrophy, cardiovascular hospitalization and mortality (15). Collectively, these findings support the crucial role of Klotho in preventing the development of cardio-renal dysfunction induced by diabetes.

Although the cardio-renal protective effects of Klotho have been well established, the predictive capabilities of serum Klotho levels for diabetes remain largely unknown. Therefore, we aimed to investigate the association between serum levels of Klotho and the prevalence of diabetes from the National Health and Nutrition Examination Survey (NHANES) 2007 to 2016 data.

## Methods

### Study population

**Figure 1:**
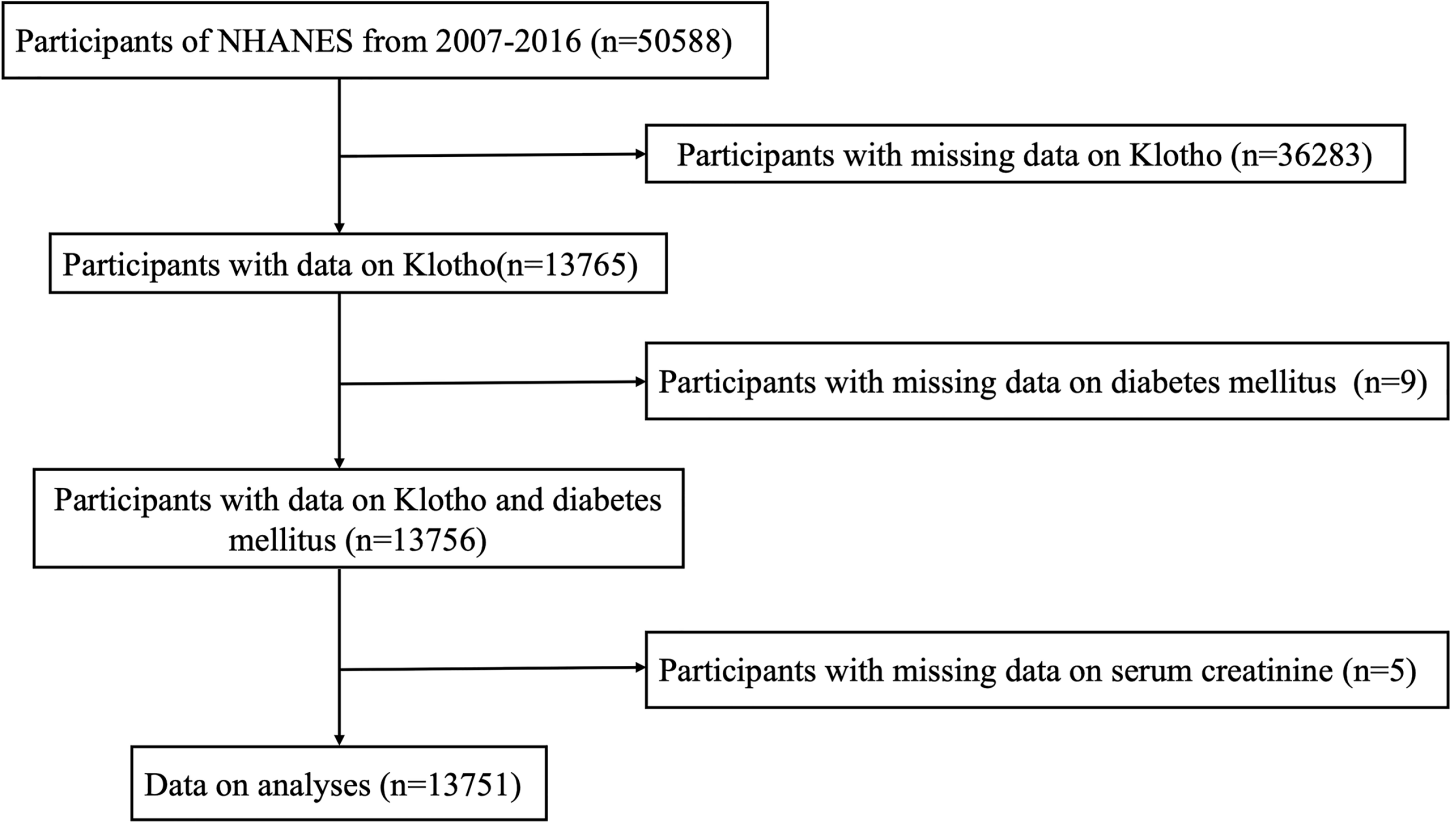
Flow chart of the study population. NHANES: the National Health and Nutrition Examination Survey.

The NHANES database can be accessed on the website of the Centers for Disease Control and Prevention National Center for Health Statistics (https://www.cdc.gov/nchs/nhanes/index.htm). Our study examined subjects participating in the 2007–2016 NHANES campaigns (n = 50588). Individuals with available data on serum Klotho concentration were included (n = 13765). A total of 13751 subjects were enrolled in the current study after further excluding participants with missing information on diabetes (n = 9) and other covariates such as serum creatinine (n = 5). The details of the participant recruitment process are illustrated in **Figure 1**. The ethical approval to conduct the NHANES 2007–2016 was granted by the NHANES Institutional Review Board (Protocol #2005-06 and Protocol #2011-17). The study procedures were structured in line with the Declaration of Helsinki. All participants provided informed consent.

### Serum Klotho measurements

The subjects were instructed to fast overnight and the blood samples were collected for analysis. The samples were collected from NHANES participants aged 40-79 years over a 10-year timeline. The sample packages were received on dry ice and inspected carefully, which were then stored at -80°C until analysis. The serum Klotho concentrations were measured using enzyme-linked immunosorbent assay (ELISA; IBL-International, Japan) and subsequently analyzed by trained statisticians (16). Following the manufacturer’s protocol, each sample analysis was repeated twice, and the average of both values was used to calculate the final result. The laboratory standards were carefully checked for acceptance of the results. All samples exceeded the 6 pg/ml limit for Klotho detection. The mean intra-assay and inter-assay coefficients of variation of Klotho measurements were 3.6% and 3.2% for the standard samples and 2.8% and 3.6% for the human samples, respectively. A more detailed description of the Klotho detection method is available on the NHANES website.

### Diabetes assessment

In this study, diabetes was defined as follows (1): a glycosylated hemoglobin A1c (HbA1c) level ≥ 6.5% (47.5 mmol/mol), or (2) fasting plasma glucose (FPG) level ≥ 126 mg/dL (7.0mmol/L), or (3) random blood glucose ≥ 200 mg/dL (11.1mmol/L), or (4) 2-hour oral glucose tolerance test (OGTT) plasma glucose ≥ 200 mg/dL (11.1mmol/L), or (5) the use of hypoglycemic drugs, or (6) participants with a self-reported diabetes diagnosis (17). Anyone who meets one of the criteria above was considered to have diabetes. Based on the NHANES population, there is a considerable overlap in the prevalence of undiagnosed diabetes as defined by FPG or HbA1c. Therefore, undiagnosed diabetes may be underestimated when using the HbA1c criteria alone (18). Despite the lack of explicit information regarding the types of diabetes in the NHANES database, this study likely enrolled a majority of type-2 diabetic (T2D) subjects since the proportion of type-1 diabetes (T1D) in adulthood is low (19).

### Covariates

The covariate selection criteria were based on biological consideration and previously published literature (20). We utilized a standardized questionnaire to obtain the demographic characteristics of each subject, including age, gender, race/ethnicity, educational level, smoking status, physical activity, alcohol consumption, history of cardiovascular disease (CVD) and hypertension. The subjects’ height, weight, and waist circumference, and body mass index (BMI) were measured and calculated during physical examination. The triglyceride (TG), total cholesterol (TC), low-density lipoprotein cholesterol (LDL-C), high-density lipoprotein cholesterol (HDL-C), and creatinine concentrations in blood samples were also evaluated.

Education level was categorized into three groups: below high school, high school, and above high school. Races and ethnicities were categorized into Mexican American, non-Hispanic black, non-Hispanic white, and other Hispanic. Smoking status was divided into three clusters: current smokers, former smokers and non-smokers. Anyone who consumed at least 12 drinks of alcohol in the last 12 months was considered an alcohol user (21). An interview was conducted with participants to determine the time they spend on physical activity during a typical week. Physical and laboratory examinations were performed following standardized procedures. BMI was calculated as weight in kilograms (kg) divided by the square of height in meters (m^2^) (22). CVD referred to congestive heart failure, coronary heart disease, angina, heart attack, and stroke as composite events. In this study, hypertension was defined by the following criteria (1): average systolic blood pressure ≥ 140 mmHg, or (2) average diastolic blood pressure ≥ 90 mmHg, or (3) the use of antihypertensive medication, or (4) subjects with a self-reported hypertension diagnosis (23). Dyslipidemia was defined as follows (1): TG ≥ 150mg/dL (1.7mmol/L), or (2)TC ≥ 200mg/dl (5.18mmol/L), or (3) LDL ≥ 130mg/dL (3.37mmol/L), or (4) HDL < 40mg/dL (1.04mmol/L) for males, HDL < 50mg/dL (1.3mmol/L) for females, or (5) the use of lipid-lowering medication (24, 25). The eGFR was calculated by using the formula developed by the Chronic Kidney Disease-Epidemiology Collaboration (CKD-EPI) (26). The NHANES website provides detailed information about these variables.

### Statistical analysis

Continuous variables were expressed as mean (standard deviation, SD) or median (interquartile range, IQR), while categorical variables were expressed as frequency (percentage). The normal distribution of continuous variables was confirmed by the histogram and Q-Q plot. Considering the skewed Klotho distributions, the data were log2-transformed to facilitate interpretation. The concentration of serum Klotho was divided into four quartiles, and the first quartile was used as the reference. When analyzing the baseline characteristics, the statistical differences between quartiles of serum Klotho were tested with ANOVA or the non-parametric test for continuous variables and the chi-square test for categorical variables.

To study the relationship between serum Klotho and diabetes, logistic generalized linear models were applied. A range of regression models (Models 1 to 3) were tested by adjusting potential confounding factors step by step. Model 1 was adjusted for age, sex, race, education, BMI, and waist circumference. Model 2 was further adjusted for smoking status, alcohol intake, physical activity, CVD, hypertension, and dyslipidemia. Model 3 was adjusted for the variables in Model 2 and additional confounders including TG, LDL-C, HDL-C, TC, and eGFR. The results were presented as adjusted odds ratios (ORs) and 95% confidence intervals (CIs). Restricted cubic spline regression was used to address the potential nonlinearity of the association between log2-transformed serum Klotho levels and diabetes. The reference was the median of log2-transformed serum Klotho levels. The association between Klotho and diabetes was assessed through subgroup analyses stratified by age, sex, BMI, CVD, hypertension, dyslipidemia, eGFR. The statistical analyses were carried out using statistical packages R version 4.1.3 (http://www.R-project.org, The R Foundation, Vienna, Austria). *P* value < 0.05 was considered statistically significant.

## Results

### Baseline characteristics of subjects

A total of 13751 subjects participated in this study, with 48.5% of them being males. The median (IQR) of serum Klotho was 802.50 (654.75, 993.25) pg/ml. The mean age ± SD of all subjects was 57.7 ± 10.9 years. The overall prevalence of diabetes was 25.9% (n = 3557), which was 27.1% (n = 933), 24.5% (n = 842), 24.7% (n = 850) and 27.1% (n = 932) in serum Klotho quartiles 1-4, respectively (*P* = 0.01). The median HbA1c levels for increasing quartiles were 5.70% (IQR 5.40%–6.10%), 5.70% (IQR 5.40%–6.00%), 5.60% (IQR 5.40%–6.10%), and 5.70% (IQR 5.40%–6.10%), respectively. Participants in the highest serum Klotho quartile were more likely to be young, female, non-Hispanic Blacks and have higher educational levels, less likely to smoke, drink alcohol, and perform physical activity, more likely to have lower waist circumference, lower levels of TG and creatinine as well as higher levels of LDL-C and HDL-C, and less likely to have dyslipidemia, CVD, and hypertension (all *P* < 0.05). In addition, participants in the higher serum Klotho quartiles was found to have higher eGFR compared to those in the lower serum Klotho quartiles, as previously reported in populations with diabetic nephropathy (DN) (13, 14). **Table 1** provides the detailed baseline information stratified by serum Klotho quartiles for all participants.

**Table 1:** Baseline characteristics of the study participants.

Variables	Overall	Serum Klotho quartiles (pg/mL)				P value
		Q1	Q2	Q3	Q4	
Participants (n)	13751	3438	3438	3437	3438	
Age (years)	57.74 (10.86)	59.14 (11.09)	57.97 (10.83)	57.39 (10.74)	56.45 (10.59)	<0.001
Sex (n, %)						<0.001
Female	7085 (51.5)	1661 (48.3)	1659 (48.3)	1809 (52.6)	1956 (56.9)	
Male	6666 (48.5)	1777 (51.7)	1779 (51.7)	1628 (47.4)	1482 (43.1)	
Education (n, %)						0.003
Below high school	1874 (13.6)	480 (14.0)	474 (13.8)	472 (13.7)	448 (13.0)	
High school	5059 (36.8)	1354 (39.4)	1240 (36.1)	1248 (36.3)	1217 (35.4)	
Above high school	6810 (49.6)	1600 (46.6)	1723 (50.1)	1714 (49.9)	1773 (51.6)	
Race, (n, %)						<0.001
Mexican American	2186 (15.9)	557 (16.2)	539 (15.7)	560 (16.3)	530 (15.4)	
Non-Hispanic Black	2723 (19.8)	698 (20.3)	567 (16.5)	585 (17.0)	873 (25.4)	
Non-Hispanic White	5918 (43.0)	1554 (45.2)	1591 (46.3)	1503 (43.7)	1270 (36.9)	
Other Hispanic	1575 (11.5)	345 (10.0)	384 (11.2)	409 (11.9)	437 (12.7)	
Other Race	1349 (9.8)	284 (8.3)	357 (10.4)	380 (11.1)	328 (9.5)	
BMI (kg/m2)	29.74 (6.71)	29.87 (6.40)	29.75 (6.64)	29.64 (6.73)	29.68 (7.05)	0.506
Waist (cm)	101.76 (15.49)	102.89 (15.03)	102.17 (15.45)	101.29 (15.37)	100.71 (16.01)	<0.001
Triglyceride (mmol/l)	1.24 (0.87, 1.82)	1.30 (0.91, 1.91)	1.28 (0.91, 1.86)	1.24 (0.87, 1.78)	1.15 (0.81, 1.69)	<0.001
Total Cholesterol (mmol/l)	5.14 (1.10)	5.14 (1.17)	5.15 (1.09)	5.14 (1.05)	5.13 (1.10)	0.906
LDL-C (mmol/l)	3.04 (0.93)	2.98 (0.97)	3.08 (0.93)	3.06 (0.93)	3.04 (0.91)	0.035
HDL-C (mmol/l)	1.37 (0.43)	1.37 (0.44)	1.36 (0.43)	1.38 (0.43)	1.39 (0.44)	0.011
Creatinine (umol/l)	75.14 (63.65, 90.17)	80.44 (65.64, 96.36)	76.91 (64.53, 90.17)	74.26 (63.65, 88.40)	72.49 (61.88, 85.75)	<0.001
Klotho (pg/mL)	802.50 (654.75, 993.25)	556.70 (489.75, 610.70)	727.50 (690.82, 763.50)	886.80 (841.60, 935.30)	1171.60 (1070.12, 1341.68)	<0.001
Smoke (n, %)						<0.001
Never	7064 (51.4)	1571 (45.7)	1701 (49.5)	1827 (53.2)	1965 (57.2)	
Former	3984 (29.0)	1091 (31.8)	1035 (30.1)	975 (28.4)	883 (25.7)	
Now	2696 (19.6)	772 (22.5)	701 (20.4)	634 (18.5)	589 (17.1)	
Alcohol intake (n, %)						<0.001
Yes	9013 (70.7)	2417 (74.9)	2301 (72.1)	2225 (70.1)	2070 (65.4)	
No	3740 (29.3)	809 (25.1)	891 (27.9)	947 (29.9)	1093 (34.6)	
CVD (n, %)						<0.001
Yes	1904 (13.8)	595 (17.3)	471 (13.7)	444 (12.9)	394 (11.5)	
No	11845 (86.2)	2842 (82.7)	2967 (86.3)	2993 (87.1)	3043 (88.5)	
Dyslipidemia (n, %)						<0.001
Yes	11002 (80.0)	2837 (82.5)	2784 (81.0)	2749 (80.0)	2632 (76.6)	
No	2749 (20.0)	601 (17.5)	654 (19.0)	688 (20.0)	806 (23.4)	
Hypertension (n, %)						<0.001
Yes	7435 (54.1)	2019 (58.7)	1842 (53.6)	1776 (51.7)	1798 (52.3)	
No	6316 (45.9)	1419 (41.3)	1596 (46.4)	1661 (48.3)	1640 (47.7)	
DM (n, %)						0.010
Yes	3557 (25.9)	933 (27.1)	842 (24.5)	850 (24.7)	932 (27.1)	
No	10194 (74.1)	2505 (72.9)	2596 (75.5)	2587 (75.3)	2506 (72.9)	
Glycosylated hemoglobin (%)	5.70 (5.40, 6.10)	5.70 (5.40, 6.10)	5.70 (5.40, 6.00)	5.60 (5.40, 6.00)	5.70 (5.40, 6.10)	0.002
eGFR (mL/min/1.73m^2^)	86.67 (20.21)	82.10 (22.68)	86.09 (19.76)	87.92 (18.43)	90.58 (18.73)	<0.001
Physical activity (never) (n, %)	8582 (62.4)	2094 (60.9)	2118(61.6)	2152 (62.6)	2218(64.6)	0.012

Data are shown as mean (SD), median (IQR), or n (%). Klotho was divided to four levels by quartile (Q1 ≤ 654.75; 654.75 < Q2 ≤ 802.50; 802.50 < Q3 ≤ 993.25; Q4 > 993.25). Abbreviations: BMI, body mass index; CVD, cardiovascular disease; DM, diabetes mellitus; LDL-C, Low-Density Lipoprotein Cholesterol; HDL-C, High density leptin cholesterol; eGFR, estimated glomerular filtration rate.

### Association between serum Klotho levels and diabetes

The logistic regression analysis results for the association between serum Klotho levels and diabetes are presented in **Table 2**. In all three regression models (Model 1-3), the increase in serum Klotho levels was positively correlated with the prevalence of diabetes. After fully adjusting for potential confounders such as age, sex, race, education, BMI, waist, smoking status, alcohol intake, physical activity, CVD, hypertension, dyslipidemia, TG, TC, LDL-C, HDL-C, and eGFR, the bottom quartile of serum Klotho levels was used as a reference, revealing that subjects in the second to the fourth quartile were at a greater risk of diabetes. The ORs with 95% CIs for diabetes in quartiles 1 to 4 were 0.96 (0.80–1.15), 0.98 (0.82–1.18), and 1.25 (1.04–1.50), respectively, in fully adjusted model (*P* for trend = 0.018).

**Table 2:** Association between serum Klotho levels and diabetes mellitus in the database from NHANES 2007-2016.

Serum Klotho quantiles (pg/ml)	Number of patients	DM (n, %)	Model 1OR (95%CI)	Model 2OR (95%CI)	Model 3OR (95%CI)
	13751	3557 (25.9)			
Q1	3438	933 (27.1)	1 (Ref).	1 (Ref).	1 (Ref).
Q2	3438	842 (24.5)	0.94 (0.83-1.06)	0.95 (0.84-1.08)	0.96 (0.80-1.15)
Q3	3437	850 (24.7)	1.04 (0.93-1.18)	1.07 (0.94-1.22)	0.98 (0.82-1.18)
Q4	3438	932 (27.1)	1.25 (1.11-1.41) ***	1.29 (1.14-1.46) ***	1.25 (1.04-1.50) *
*P* for trend			<0.001	<0.001	0.018

Model 1 was adjusted for age, sex, race, education, BMI, and waist.

Model 2 was adjusted for Model 1 + smoking status, alcohol intake, physical activity, CVD, dyslipidemia, hypertension.

Model 3 was adjusted for Model 2 + triglyceride, LDL-C, HDL-C, total Cholesterol and eGFR.

NHANES: the National Health and Nutrition Examination Survey; BMI, body mass index; CVD, cardiovascular disease; DM, diabetes mellitus; LDL-C, Low-Density Lipoprotein Cholesterol; HDL-C, High density leptin cholesterol; eGFR, estimated glomerular filtration rate; OR, Odds Ratio; CI, Confidence Interval.

***P < 0.001, *P < 0.05.

**Figure 2:**
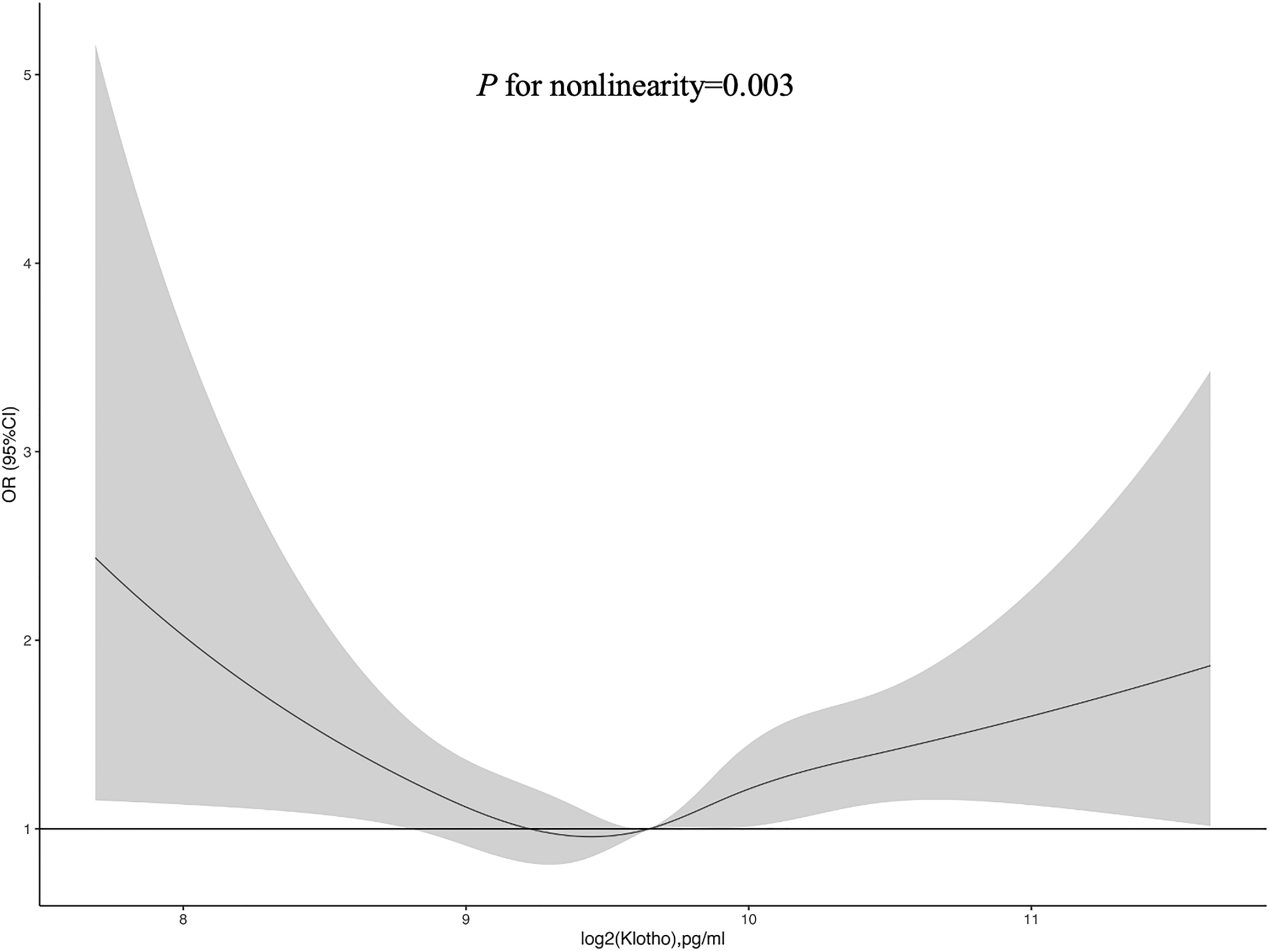
Restricted cubic spline of the association between serum Klotho and diabetes. The association was adjusted for age, sex, educational level, race, BMI, waist circumference, smoking status, alcohol intake, physical activity, CVD, hypertension, dyslipidemia, total cholesterol, LDL-C, HDL-C, triglyceride, and eGFR. The median log2-transformed serum Klotho level was chosen as a reference. Abbreviations: BMI, body mass index; CVD, cardiovascular disease; DM, diabetes mellitus; LDL-C, Low-Density Lipoprotein Cholesterol; HDL-C, High density leptin cholesterol; eGFR, estimated glomerular filtration rate.

**Figure 2** exhibited the restricted cubic spline plot for analyzing the association between log2-transformed serum Klotho levels and the risk of diabetes, demonstrating a U-shaped relationship (*P* for nonlinearity = 0.003). When log2-transformed serum Klotho level ≤ median, the decreasing prevalence of diabetes was associated with increasing Klotho level. Conversely, when log2-transformed serum Klotho levels were higher than the median value, increasing Klotho levels were associated with a higher prevalence of diabetes.

### Subgroup analysis

**Figure 3:**
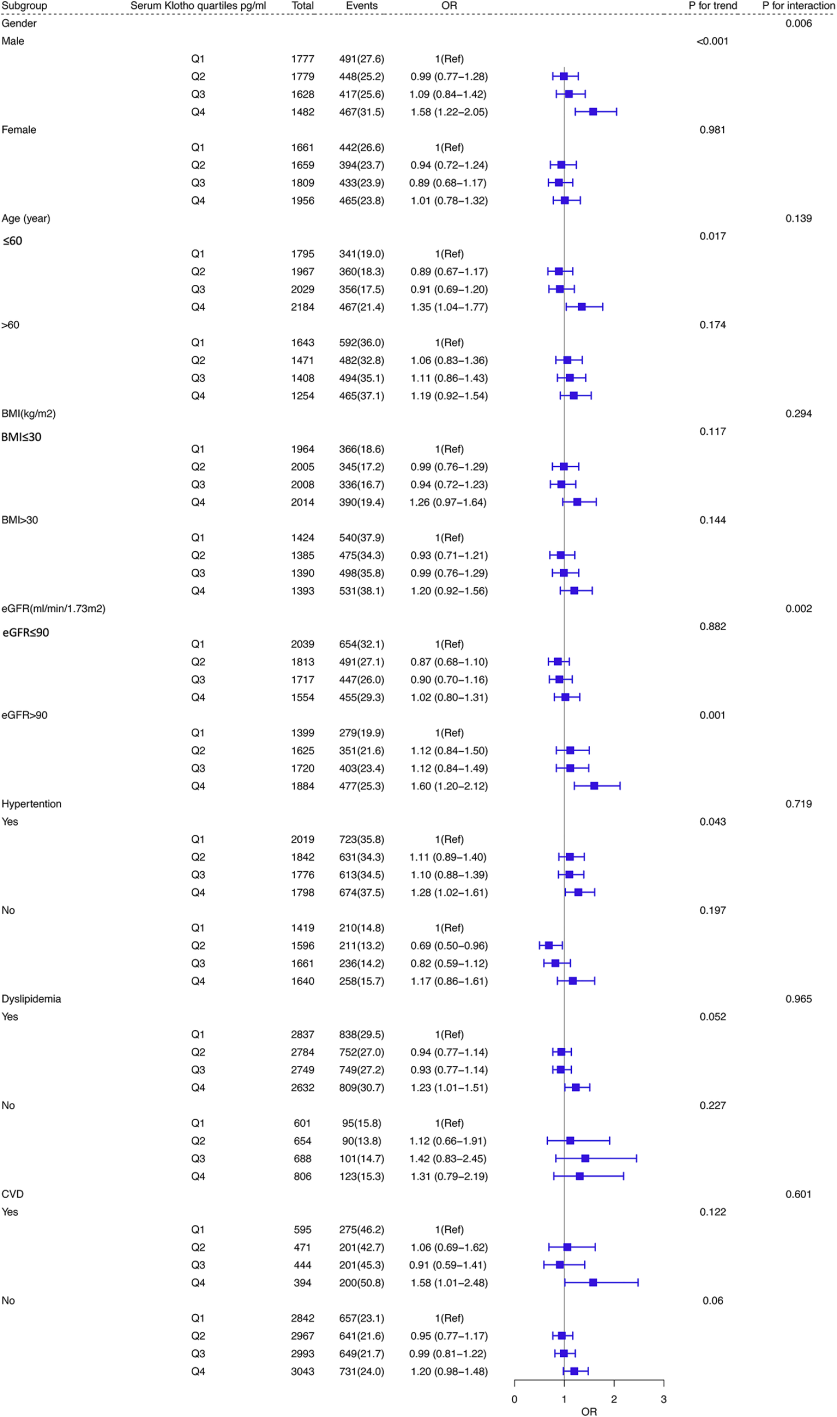
Subgroup analyses of the association between serum Klotho levels and DM in the database from NHANES 2007–2016. Adjusted for age, sex, educational level, race, BMI, waist circumference, smoking status, alcohol intake, physical activity, CVD, hypertension, dyslipidemia, total cholesterol, LDL-C, HDL-C, triglyceride, and eGFR. Abbreviations: NHANES: the National Health and Nutrition Examination Survey; BMI, body mass index; CVD, cardiovascular disease; DM, diabetes mellitus; LDL-C, Low-Density Lipoprotein Cholesterol; HDL-C, High density leptin cholesterol; eGFR, estimated glomerular filtration rate.

As shown in **Figure 3**, subgroup analysis was performed based on age, sex, BMI, dyslipidemia, eGFR and history of CVD and hypertension. Statistically significant interactions were observed between Klotho levels and sex and eGFR (*P* for interaction < 0.05). Participants who were male, younger than 60 years old, had a history of hypertension and a higher level of eGFR (≥ 90 ml/min/1.73 m^2^) were inclined to have a higher risk of diabetes in the highest quartile (Q4) of Klotho than those in Q1 (all *P* for trend < 0.05).

## Discussion

Our study is the first to detect the association of Klotho with diabetes in a large, unselected population. Serum Klotho levels are nonlinearly and positively correlated with the prevalence of diabetes, independent of other confounding factors, including age, sex, race, education, BMI, waist, smoking status, alcohol intake, physical activity, CVD, hypertension, dyslipidemia, TG, TC, LDL-C, HDL-C, and eGFR. In this large, cross-sectional study, data from 13751 US civilians in NHANES 2007–2016 were collected and analyzed. The prevalence of diabetes increased with increasing quartiles of serum Klotho. In the fully adjusted model, the risk of diabetes for subjects in the top quartile was 1.25 times higher than those in the bottom quartile.

Klotho has been demonstrated to exert therapeutic effects on diabetes and its complications. Klotho^-/-^ mutant mice display severe pancreatic islet atrophy, downregulated mRNA and protein levels of insulin in pancreatic islets, as well as reduced serum insulin levels, suggesting that disturbance in Klotho signaling leads to dysregulation of the glucose homeostasis (27). There is an abundant expression of Klotho in the insulinoma β cells in mouse pancreatic islets (MIN6 β cells), and its silencing/overexpression significantly reduces/increases glucose-stimulated insulin production in MIN6 β cells (28). Downregulated Klotho expression was consistently observed in pancreatic islet β cells of diabetic human patients and in a rodent model of diabetes (db/db mice). It is strongly related to a decrease in insulin secretion from pancreatic β-cells. β cell-specific expression of Klotho preserved the β cell function, ameliorated hyperglycemia, enhanced glucose tolerance and prevented the development of diabetes in db/db mice. In this process, Klotho may exert beneficial effects *via* stimulating β cell proliferation, alleviating oxidative stress and endoplasmic reticulum (ER) stress, inhibiting apoptosis as well as normalizing autophagy (29). Besides, both *in vitro* and *in vivo* evidence have demonstrated that Klotho can attenuate diabetic nephropathy and cardiomyopathy, indicating the cardio-renal protective effects of Klotho (8–11).

It is noteworthy that previous evidence concerning the changes in serum Klotho levels in the context of diabetes is contradictory. Several studies have demonstrated that serum levels of Klotho in individuals with either T2D or T1D were significantly lower compared to those of healthy controls (30–34). In cultured human embryonic kidney epithelial cells HEK293 and human renal proximal tubular epithelial cells HK2, high glucose exposure markedly downregulated the protein and mRNA level of Klotho in a time-dependent manner (32). In line with these findings, a meta-analysis of 14 unique studies indicated a decrease in serum Klotho levels in diabetic patients rather than those without diabetes, and such decrease was more remarkable in patients with DN, especially during the early stages of DN (35). In contrast, some researchers have reported that serum Klotho levels are greatly upregulated in patients with diabetes compared with the levels in healthy controls (36, 37). Lee et al. reported higher serum Klotho levels in diabetic patients with relatively preserved renal function than in healthy subjects, and decreased with exacerbating albuminuria. These findings suggested that Klotho may be a novel and useful early biomarker of diabetic renal injury (38). A recent study also found that diabetic patients with poorer glycemic control had higher serum Klotho concentrations, especially if their renal function was preserved, which is completely consistent with our findings (39). Interestingly, there was another study detecting similar circulating Klotho levels between patients with T2D and healthy individuals in the absence of DN (40). Taken together, the discrepancies among previous publications can be largely attributed to the dynamic changes in Klotho expression at different stages of diabetes. This explanation was consistent with the findings from another study that the serum levels of Klotho increased at the onset of diabetes as a potential compensatory mechanism in response to high glucose stimulation, while their levels recovered as diabetes progressed (41). More importantly, considering that kidneys act as a major source of endogenous Klotho and the presence of elevated Klotho levels is highly related to preserved renal function (42), we may speculate that serum Klotho concentration serves as a biomarker of the number of functional nephrons and therefore reflects the degree of renal impairment induced by high glucose. In this regard, further studies are needed to clarify the exact causal relationship between diabetes and Klotho levels.

Glycosylated hemoglobin has been established as a reliable index of diabetic blood glucose control soon after its discovery in the 1960s (43), with HbA1c being the most abundant hemoglobin component in human erythrocytes (44). Once HbA1c was formed inside the body, it may persist stably throughout the whole lifespan of the erythrocytes (approximately 120 days) and reflect the dynamic changes of blood glucose concentration, as demonstrated by *in vitro* studies that the rate of formation of HbA1c increased proportionately with increasing glucose concentration (45). The cutoff value for the HbA1c is typically set to 6.5% when it is used for diagnosis of diabetes. There were significantly reduced Klotho levels in blood samples of diabetic patients (HbA1c ≥ 6.5%) compared with control samples (HbA1c < 6.5%) (46). Despite an inverse association between serum Klotho level and HbA1c has been found in both T1D and T2D patients by several studies (32–34); however, no significant association (47) or even a positive association (39) between the two has also been reported. Given Klotho may also serve as a potential predictive marker for diabetes, future studies are needed to shed light on the explicit association of serum Klotho level with HbA1c and reveal the underlying mechanisms.

The main strength of our study is the robust evidence on the association of serum Klotho levels with diabetes based on a nationally representative and noninstitutionalized US civilian population. The NHANES is a large-scale study that employs standardized study protocols, strict quality control metrics, and well-trained specialized technicians to collect and process data. Being based on the NHANES database, the large sample size in our study ensured high statistical power to perform subgroup analyses and assess the impact of a series of predictors in multivariate models. Our research discovered a nonlinear and positive association between serum Klotho levels and diabetes, independent of other confounding factors with known effects on the development of diabetes, including such as educational level, race, BMI, waist circumference, smoking status, alcohol intake, physical activity, history of CVD and hypertension, dyslipidemia, TG, LDL-C, HDL-C, TC, and eGFR. All adjusted models confirmed the robustness of this association. Considering the strong association of diabetes with aging and the anti-aging property of Klotho, a lower prevalence of diabetes appears to be more reasonable as serum Klotho concentrations increase. A possible explanation for this serendipity may be related to the increased metabolic demands in the distal and proximal kidney tubules caused by glycosuria, which may lead to increased Klotho production as well as higher serum Klotho levels. In fact, the expression levels of Klotho in cultured human tubular epithelial cells were not altered when exposed to a high glucose environment *in vitro* (40), which suggests that the intact nephron may demonstrate paracrine and endocrine effects in response to high glucose stimulation.

However, our study has several limitations. Firstly, a causal relationship cannot be inferred owing to the cross-sectional nature of the study. Future longitudinal investigations are required to determine the predictive value of Klotho in the context of diabetes. Secondly, although we have attempted to search the literature and adjust for potential confounders as much as possible, there still exists some unknown or unmeasured confounders that may also play a role in the pathogenesis of diabetes. Thirdly, although explicit information regarding the types of diabetes in the NHANES database is lacking, our study sample is representative of a general adult population. It is likely that the vast majority of subjects in the analysis are T2D patients. However, there is a considerable overlap in the prevalence of undiagnosed diabetes as defined by FPG or HbA1c separately (18). In addition, although self-reporting is the most widely-utilized method to evaluate the presence of diabetes (and other covariates) in epidemiological surveys, the self-reported information collected by questionnaires may involve recall bias due to the retrospective nature of the self-report measures. Finally, given that changes in Klotho concentrations were not considered in the NHANES study, our results can only reflect the positive association between Klotho and diabetes during the initial phase of the study. Therefore, the significance of long-term Klotho concentrations in predicting the risk of diabetes remains unelucidated.

## Conclusion

In summary, our study demonstrated that serum Klotho levels were nonlinearly and positively associated with the prevalence of diabetes in a nationally representative adult population. Prospective and mechanistic studies are necessary to elucidate the causality of the association.

## Data availability statement

The datasets presented in this study can be found in online repositories. The names of the repository/repositories and accession number(s) can be found below: https://www.cdc.gov/nchs/nhanes/index.htm.

## Ethics statement

The ethical approval to conduct the NHANES 2007-2016 was granted by the NHANES Institutional Review Board (Protocol #2005-06 and Protocol #2011-17). The study procedures were structured in line with the Declaration of Helsinki. All participants provided informed consent. The patients/participants provided their written informed consent to participate in this study.

## Author contributions

KW acquired and analyzed the data as well as drafted the original manuscript. All authors substantially contributed to the conception, design, interpretation, and critical revisions of this manuscript. All authors contributed to the article and approved the submitted version.

## Funding

This work was supported by a grant from National Natural Science Foundation of China (No. 81871359 and No. 82071944) and Natural Science Foundation of Jiangsu Province (No. BK20191496).

## Conflict of interest

The authors declare that the research was conducted in the absence of any commercial or financial relationships that could be construed as a potential conflict of interest.

## Publisher’s note

All claims expressed in this article are solely those of the authors and do not necessarily represent those of their affiliated organizations, or those of the publisher, the editors and the reviewers. Any product that may be evaluated in this article, or claim that may be made by its manufacturer, is not guaranteed or endorsed by the publisher.
